# Patient and hospital characteristics predict prolonged emergency department length of stay and in-hospital mortality: a nationwide analysis in Korea

**DOI:** 10.1186/s12873-022-00745-y

**Published:** 2022-11-21

**Authors:** Kyung-Shin Lee, Hye Sook Min, Jae Young Moon, Daesung Lim, Younghwan Kim, Eunsil Ko, You Sun Kim, Joohae Kim, Jeehye Lee, Ho Kyung Sung

**Affiliations:** 1 Public Health Research Institute, National Medical Center, 245 Eulgi-Ro, Jung-Gu, Seoul, 04564 Korea; 2 Department of Internal Medicine, Chungnam National University School of Medicine, Daejeon, Korea; 3 Department of Pulmonary and Critical Care Medicine, Chungnam National University Sejong Hospital, Sejong, Korea; 4 Department of Emergency Medicine, Seoul Medical Center, Seoul, Korea; 5 Department of Trauma Surgery, National Medical Center, Seoul, Korea; 6 National Emergency Medical Center, National Medical Center, Seoul, Korea; 7 Department of Pediatrics, National Medical Center, Seoul, Korea; 8 Division of Pulmonary and Critical Care Medicine, Department of Internal Medicine, National Medical Center, Seoul, Korea

**Keywords:** Emergency department, Critical care, Intensive care unit, Length of stay, In-hospital mortality

## Abstract

**Background:**

Prolonged emergency department length of stay (EDLOS) in critically ill patients leads to increased mortality. This nationwide study investigated patient and hospital characteristics associated with prolonged EDLOS and in-hospital mortality in adult patients admitted from the emergency department (ED) to the intensive care unit (ICU).

**Methods:**

We conducted a retrospective cohort study using data from the National Emergency Department Information System. Prolonged EDLOS was defined as an EDLOS of ≥ 6 h. We constructed multivariate logistic regression models of patient and hospital variables as predictors of prolonged EDLOS and in-hospital mortality.

**Results:**

Between 2016 and 2019, 657,622 adult patients were admitted to the ICU from the ED, representing 2.4% of all ED presentations. The median EDLOS of the overall study population was 3.3 h (interquartile range, 1.9–6.1 h) and 25.3% of patients had a prolonged EDLOS. Patient characteristics associated with prolonged EDLOS included night-time ED presentation and Charlson comorbidity index (CCI) score of 1 or higher. Hospital characteristics associated with prolonged EDLOS included a greater number of staffed beds and a higher ED level. Prolonged EDLOS was associated with in-hospital mortality after adjustment for selected confounders (adjusted odds ratio: 1.18, 95% confidence interval: 1.16–1.20). Patient characteristics associated with in-hospital mortality included age ≥ 65 years, transferred-in, artificially ventilated in the ED, assignment of initial triage to more urgency, and CCI score of 1 or higher. Hospital characteristics associated with in-hospital mortality included a lesser number of staffed beds and a lower ED level.

**Conclusions:**

In this nationwide study, 25.3% of adult patients admitted to the ICU from the ED had a prolonged EDLOS, which in turn was significantly associated with an increased in-hospital mortality risk. Hospital characteristics, including the number of staffed beds and the ED level, were associated with prolonged EDLOS and in-hospital mortality.

**Supplementary Information:**

The online version contains supplementary material available at 10.1186/s12873-022-00745-y.

## Background

Over the past few decades, critical care has become a significant and growing part of the treatment provided at emergency departments (EDs) [1, 2]. Studies conducted in the United States (US) reported that intensive care unit (ICU) admissions from the ED have increased at a greater rate than overall ED presentations, and that length of stay in the ED has markedly increased [3, 4]. The increasing provision of critical care in EDs contributes to hospital overcrowding and strain on emergency care systems, which is an important public health concern worldwide [5–7].

Critical care is extremely resource-intensive, and often requires extensive diagnostic testing, continuous monitoring, and invasive techniques [8, 9]. However, EDs are essentially designed to provide rapid triage, stabilization, and initial treatment for numerous patients with various conditions and acuity. Therefore, EDs may not be sufficiently equipped or staffed to provide the complex and continuous care needed for critically ill patients [2]. In addition, physicians and nurses in overcrowded EDs may not be able to provide timely care to critically ill patients [10, 11]. Therefore, there is a potential advantage in transferring critically ill patients immediately after stabilization from the ED to the ICU, which is a highly specialized and skilled setting for critical care [12].

ED length of stay (EDLOS), defined as the time interval from when a patient arrives at the ED until the patient leaves the ED, is a widely adopted performance indicator in studies evaluating ED processes [13, 14]. Prolonged EDLOS is associated with inefficient ED organization, untimely care, and poor adherence to clinical guidelines [15–20]. EDLOS has also been used as a proxy for ED overcrowding and boarding, which are potential threats to patient safety [21, 22]. Prolonged EDLOS in critically ill patients is associated with adverse outcomes, including increased mortality risk [23–28].

Previous studies have demonstrated the contribution of patient characteristics as predictors of prolonged EDLOS and the resulting outcome. However, Chalfin et al. suggested that certain institutional and structural factors may have contributed to these differences [25]. In fact, a study using health data from Ontario, Canada, indicated that the demand and capacity of ED and ICU were important determinants of prolonged EDLOS in critically ill patient [29]. However, since most other studies examining both patient and hospital factors have been limited to a single or selected hospitals, these results have limited generality [15, 18, 30–32].

Therefore, this nationwide study aimed to provide insight into the patient and hospital characteristics associated with prolonged EDLOS in critically ill patients directly admitted from the ED to the ICU. The secondary objective was to explore the association between prolonged EDLOS and patient outcomes, as well as related patient and hospital characteristics.

## Methods

### Study design

This study used data from a health database in Korea, the National Emergency Department Information System (NEDIS), between 2017 and 2019. The NEDIS is a nationwide ED-based database for evaluating the emergency care system in Korea, established in accordance with Article 15 of the Emergency Medical Service Act. To achieve this goal, the NEDIS collects ED visit-level data, including demographic, clinical, and administrative information. Each visit-level datum also has the corresponding hospital identifier and hospital characteristics, such as total staffed beds, level of ED, and region. All patient-related information was anonymized and electronically submitted to the central processing facility, which was examined both manually and using computerized algorithms to detect data inconsistencies. Between 2017 and 2019, the participation rate of nationwide EDs in the NEDIS was 99.3% (413/416) in 2017, 99.5% (399/401) in 2018, and 99.8% (401/402) in 2019. The design and variables of the NEDIS database have been described elsewhere [33–35].

From the NEDIS database, we identified all patients admitted to the ICU directly from the ED between 1 January 2017 and 31 December 2019 based on the date of presentation to the ED. In Korea, there are several types of ICUs that provide intensive and specialized medical and nursing care to critically ill patients with various conditions [36]. However, in this study, the ICU was defined as any type of licensed ICU within the hospital. These operational definitions for critically ill patient and ICU were adopted from previous studies [3, 6, 29]. Patients with missing age or sex information, those < 18 years old, and those with missing days and times of ED presentation and departures were excluded.

### Outcomes and variables

The primary outcome of this study was prolonged EDLOS, which was defined as an EDLOS of 6 h or more. This decision was based on existing evidence suggesting that an EDLOS of 6 h or more is associated with increased mortality risk and influences the quality of care in critically ill patients in the ED [25, 37, 38]. The secondary outcome was in-hospital mortality.

We identified patient and hospital variables a priori as potential predictors of prolonged EDLOS and in-hospital mortality risk in critically ill patients. Potential predictors were selected based on a review of the academic literature and data available in the NEDIS database [23–28, 39–42].

Patient variables included age, sex, insurance type, injury code, emergency ambulance attendance, transferred-in, date and time of ED presentation, initial triage score, artificial ventilation in the ED, diagnosis codes during hospitalization, Charlson comorbidity index (CCI), and discharge status. The initial triage was scored according to the Korean Triage and Acuity Scale (KTAS), which prioritizes patients according to the five ordinal scales reflecting clinical severity and acuity as follows: resuscitation: 1, emergent: 2, urgent: 3, less urgent: 4, non-urgent: 5 [43]. In Korea, the triage process begins with the patient’s ED presentation and can only be performed by qualified physicians, nurses, or paramedics [44]. The date and time of ED presentation were categorized according to the year, season (spring: March–May, summer: June–August, fall: September–November, winter: December–February) and ED shift time (day: 07:00–14:59, evening: 15:00–22:59, night: 23:00–06:59). Diagnostic codes used during hospitalization were identified based on codes defined in the International Classification of Diseases, Tenth Revision (ICD-10). The CCI score was calculated based on diagnostic codes used during hospitalization by applying the methods proposed in previous studies, which showed good-to-excellent discriminant power in predicting in-hospital mortality risk [45, 46].

Hospital variables were hospital staffed beds (≥ 1,000, 800–999, 600–799, 300–599, and < 300), level of ED (levels 1, 2, and 3), and location (metropolitan city versus provincial area). In Korea, EDs are classified into three levels in the following order according to their capabilities and functions: level 1, regional emergency centers; level 2, local emergency centers; and level 3, local emergency institutes [47]. Level 1 is the highest, with more medical staff, wider care space, and more equipment according to the standards specified by the Ministry of Health and Welfare.

### Statistical analysis

We calculated the proportion of the study population with overall ED presentations and overall adult ED presentations, as well as the annual incidence/100,000 adult ED presentations.

Descriptive analyses were performed to compare the patient and hospital characteristics between critically ill patients with an EDLOS of ≥ 6 h and critically ill patients with an EDLOS of < 6 h. Categorical variables were reported as frequencies and proportions and were compared between patient groups using Pearson’s Chi-squared test. Continuous variables were described as the median and interquartile range (IQR) and were tested using the Wilcoxon rank-sum test. The median EDLOS with IQR and percentages of in-hospital mortality for each of the most common primary diagnoses were calculated.

We performed multivariate logistic regression analyses to model the effects of patient and hospital variables as predictors of prolonged EDLOS (both 6 h and 12 h) and in-hospital mortality. Since level 3 EDs may differ from level 1 or 2 EDs in ED settings, patient populations, and disease spectra [48], we conducted sensitivity analyses excluding patients from level 3 EDs using the same model. To evaluate the potential differential associations of hospital characteristics with prolonged EDLOS vs. in-hospital mortality, we performed stratified analyses with the highest hospital staffed bed category (1,000 or more) or type of ED (level 1) as the reference in the same logistic regression model.

All analyses were performed using SAS version 9.4 (SAS Inc., Cary, NC, USA) and R version 4.1.3 (R Development Core Team, https://cran.r-project.org/). All tests were two-tailed, and a *p*-value < 0.05 was considered statistically significant.

## Results

### Characteristics of critically ill patients directly admitted to the intensive care unit from the emergency department

**Fig. 1:**
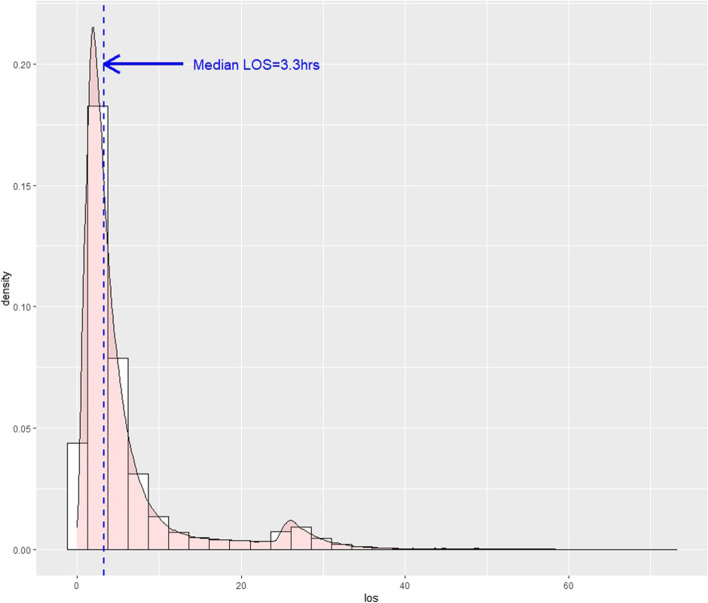
Distribution of emergency department length of stay in critically ill patients directly admitted to the intensive care unit from the emergency department. Abbreviations: EDLOS, emergency department length of stay; IQR, interquartile range

Over the 3-year study period, 657,622 adult patients directly admitted to the ICU through the ED were identified in the NEDIS database, representing 3.0% of all adults presenting to the EDs and 2.4% of all ED presentations. The crude incidence rate/100,000 adult ED presentations was 3,026 in 2017, 2,955 in 2018, and 3,048 in 2019. Of the overall study population, 166,528 (25.3%) were transferred to the ICU after a stay of ≥ 6 h in the ED, and 491,094 (74.7%) were transferred from the ED to the ICU for < 6 h in the ED. The median EDLOS of the overall study population was 3.3 h (IQR, 1.9–6.1 h) (Fig. 1).

Compared to critically ill patients with an EDLOS of < 6 h, those with an EDLOS of 6 h or more had a higher proportion of night-time presentations and had higher CCI scores (Table 1). Regarding hospital variables, an EDLOS of ≥ 6 h correlated with a greater number of staffed beds and a higher proportion of presentations to level 1 EDs than did EDLOS < 6 h. The characteristics of the study population, stratified according to staffed-bed category and type of ED, are shown in Additional file 1: Tables S1 and S2. In brief, the higher the staffed bed category and ED level, the greater the number of critically ill patients per institution, and the longer the median EDLOS.

**Table 1:** Characteristics of critically ill patients directly admitted to the ICU from the ED according to EDLOS

	Overall (*n* = 657,622)	EDLOS < 6 h (*n* = 491,094)	EDLOS ≥ 6 h (*n* = 166,528)	*P* value
Patient variables				
Age ≥ 65 y	386,687 (58.8)	287,282 (58.5)	99,405 (59.7)	< 0.0001
Median (IQR)	69 (56–80)	69 (56–80)	70 (57–79)	0.110
Female	268,064 (40.8)	200,284 (40.8)	67,780 (40.7)	0.559
Insurance type				
National health insurance	536,123 (81.5)	398,242 (81.1)	137,881 (82.8)	< 0.0001
Medical aid	81,099 (12.3)	59,750 (12.2)	21,349 (12.8)	
Uninsured or other	40,400 (6.2)	33,102 (6.7)	7,298 (4.4)	
Injury-related presentation	99,695 (15.2)	80,216 (16.3)	19,479 (11.7)	< 0.0001
Arrival via emergency ambulance	283,399 (43.1)	214,615 (43.7)	68,784 (41.3)	< 0.0001
Transferred-in	230,098 (35.0)	168,167 (34.2)	61,931 (37.2)	< 0.0001
Time of presentation				
Day	286,545 (43.6)	218,704 (44.5)	67,841 (40.7)	< 0.0001
Evening	264,295 (40.2)	198,895 (40.5)	65,400 (39.3)	
Night	106,782 (16.2)	73,495 (15.0)	33,287 (20.0)	
KTAS score				
1	60,253 (9.2)	42,731 (8.7)	17,522 (10.5)	< 0.0001
2	217,028 (33.0)	161,540 (32.9)	55,488 (33.3)	
3	266,146 (40.5)	190,774 (38.8)	75,372 (45.3)	
4	49,610 (7.5)	36,499 (7.4)	13,111 (7.9)	
5	6,928 (1.1)	5,431 (1.2)	1,497 (0.9)	
Unidentified	57,657 (8.7)	54,119 (11.0)	3,538 (2.1)	
Artificial ventilation in the ED	37,236 (5.7)	27,491 (5.6)	9,745 (5.9)	0.0001
CCI score				
0	442,997 (67.4)	340,937 (69.4)	102,060 (61.3)	< 0.0001
1	46,805 (7.1)	33,096 (6.7)	13,709 (8.2)	
2	112,117(17.0)	79,477 (16.2)	32,640 (19.6)	
≥ 3	55,703 (8.5)	37,584 (7.7)	18,119 (10.9)	
Season				
Spring	164,752 (25.1)	122,763 (25.0)	41,989 (25.2)	< 0.0001
Summer	165,635 (25.2)	124,844 (25.4)	40,791 (24.5)	
Fall	166,611 (25.3)	125,244 (25.5)	41,367 (24.8)	
Winter	160,624 (24.4)	118,243 (24.1)	42,381 (25.4)	
Year				
2017	216,632 (32.9)	164,451 (33.5)	52,181 (31.3)	< 0.0001
2018	218,519 (33.2)	161,159 (32.8)	57,360 (34.4)	
2019	222,471 (33.8)	165,484 (33.7)	56,987 (34.2)	
Hospital variables				
Hospital staffed beds				
≥ 1,000	113,419 (17.2)	66,518 (13.5)	46,901 (28.2)	< 0.0001
800–999	155,278 (23.6)	103,283 (21.0)	51,995 (31.2)	
600–799	128,668 (19.6)	89,003 (18.1)	39,665 (23.8)	
300–599	152,294 (23.2)	130,977 (26.7)	21,317 (12.8)	
< 300	107,963 (16.4)	101,313 (20.7)	6,650 (4.0)	
Level of ED				
Level 1	253,879 (38.6)	169,283 (34.5)	84,596 (50.8)	< 0.0001
Level 2	298,988 (45.5)	224,332 (45.7)	74,656 (44.8)	
Level 3	104,755 (15.9)	97,479 (19.8)	7,276 (4.4)	
Location				
Metropolitan city	305,370 (46.4)	216,327 (44.1)	89,043 (53.5)	< 0.0001
Provincial area	352,252 (53.6)	274,767 (55.9)	77,485 (46.5)	

Data are presented as number (%), unless otherwise indicated

*ICU* Intensive care unit, *ED* Emergency department, *EDLOS* Emergency department length of stay, *IQR* Interquartile range, *KTAS* Korean triage and acuity scale, *CCI* Charlson comorbidity index

The most common primary diagnosis in the study population was acute myocardial infarction, accounting for 8.6% of adult patients directly admitted to the ICU from the ED, with a median EDLOS of 2.1 h (IQR, 0.9–4.9 h), and an in-hospital mortality rate of 7.6%. The next most common primary diagnoses were intracranial injury (7.2%), cerebral infarction (7.0%), pneumonia (5.4%), and intra-cerebral hemorrhage (4.6%) (Table 2).

**Table 2:** The ten most common primary diagnoses for critically ill patients directly admitted to the ICU from the ED

	Primary diagnosis (ICD-10 code)	n (%)	Median EDLOS, h (IQR)	In-hospital mortality (%)
1	Acute myocardial infarction (I21)	56,328 (8.6)	2.1 (0.9–4.9)	4,287 (7.6)
2	Intracranial injury (S06)	47,114 (7.2)	2.5 (1.6–4.2)	6,297 (13.4)
3	Cerebral infarction (I63)	46,138 (7.0)	3.0 (1.9–5.0)	3,652 (7.9)
4	Pneumonia (J18)	35,803 (5.4)	3.5 (2.2–6.2)	9,920 (27.7)
5	Intracerebral hemorrhage (I61)	30,100 (4.6)	2.3 (1.5–3.8)	5,084 (16.9)
6	Subarachnoid hemorrhage (I60)	17,998 (2.7)	2.4 (1.6–3.7)	3,161 (17.6)
7	Heart failure (I50)	17,660 (2.7)	4.0 (2.4–7.5)	2,433 (13.8)
8	Other sepsis (A41)	12,707 (1.9)	4.5 (2.8–7.8)	4,262 (33.5)
9	Angina pectoris (I20)	11,206 (1.7)	3.4 (1.7–6.3)	168 (1.5)
10	Cardiac arrest (I46)	9,625 (1.5)	3.2 (1.8–5.9)	4,597 (47.8)

*ICU* Intensive care unit, *ED* Emergency department, *ICD-10* International classification of diseases 10^th^, *EDLOS* Emergency department length of stay, *IQR* Interquartile range

### Variables associated with prolonged emergency department length of stay

The results of multivariate logistic regression analyses with prolonged EDLOS as the dependent outcome are shown in Table 3. For the patient variables of interest, night-time ED presentation was a significant predictor of prolonged EDLOS (adjusted odds ratio [aOR], 1.49; 95% confidence interval (CI), 1.46–1.51). KTAS scores of 4 (aOR, 1.48; 95% CI, 1.44–1.53) and 5 (aOR, 1.39; 95% CI, 1.30–1.48), indicating lower acuity, were significant predictors of prolonged EDLOS compared with a KTAS score 1 of the highest acuity. In addition, a CCI score of 1 or higher significantly predicted an EDLOS of ≥ 6 h, with patients with a CCI score of zero as the reference. For the hospital variables of interest, hospital staffed bed category was a significant predictor of prolonged EDLOS. Critically ill patients who admitted to hospitals with < 300 staffed beds were less likely to have prolonged EDLOS than those who admitted to hospitals with ≥ 1,000 staffed beds (aOR, 0.12; 95% CI, 0.11–0.12). These findings were consistent with those at the ED level. In the sensitivity analysis, the magnitude and direction of the aOR of patient and hospital variables did not change after excluding patients presented in the level 3 ED (Additional file 1: Table S3).

**Table 3:** Multivariate logistic regression analyses for prolonged EDLOS

	EDLOS ≥ 6 h		EDLOS ≥ 12 h	
	aOR	95% CI	aOR	95% CI
Patient variable				
Age ≥ 65 y (vs. < 65 y)	1.16	1.14–1.17	1.09	1.07–1.11
Female (vs. male)	1.02	1.01–1.03	1.00	0.99–1.02
Insurance type				
National health insurance	1.00	(Reference)	1.00	(Reference)
Medical aid	1.27	1.25–1.29	1.24	1.21–1.27
Uninsured or other	0.83	0.81–0.86	0.82	0.79–0.86
Injury-related presentation (vs. no)	0.64	0.63–0.65	0.69	0.67–0.71
Arrival via emergency ambulance (vs. other)	1.00	0.99–1.02	0.98	0.96–1.00
Transferred-in (vs. direct)	0.92	0.91–0.94	1.02	1.00–1.04
Time of presentation				
Day	1.00	(Reference)	1.00	(Reference)
Evening	1.09	1.07–1.10	3.49	3.43–3.55
Night	1.49	1.46–1.51	2.69	2.63–2.76
KTAS score				
1	1.00	(Reference)	1.00	(Reference)
2	0.87	0.85–0.89	0.86	0.84–0.89
3	1.12	1.10–1.15	1.00	0.97–1.03
4	1.48	1.44–1.53	1.25	1.20–1.30
5	1.39	1.30–1.48	1.15	1.06–1.26
Unidentified	1.01	0.96–1.06	1.00	0.93–1.07
Artificial ventilation in the ED (vs. no)	0.89	0.87–0.91	0.94	0.91–0.97
CCI score				
0	1.00	(Reference)	1.00	(Reference)
1	1.37	1.34–1.40	1.29	1.25–1.33
2	1.30	1.28–1.32	1.25	1.22–1.27
≥ 3	1.46	1.43–1.49	1.35	1.32–1.39
Season				
Spring	1.00	(Reference)	1.00	(Reference)
Summer	0.94	0.92–0.96	0.91	0.89–0.93
Fall	0.95	0.93–0.96	0.92	0.90–0.94
Winter	1.05	1.03–1.07	1.10	1.08–1.13
Year				
2017	1.00	(Reference)	1.00	(Reference)
2018	1.10	1.09–1.12	1.10	1.08–1.12
2019	1.05	1.03–1.06	1.00	0.98–1.02
Hospital variables				
Hospital staffed beds				
≥ 1,000	1.00	(Reference)	1.00	(Reference)
800–999	0.69	0.68–0.70	0.81	0.79–0.83
600–799	0.63	0.62–0.64	0.72	0.70–0.74
300–599	0.23	0.23–0.24	0.28	0.28–0.29
< 300	0.12	0.11–0.12	0.19	0.18–0.20
Level of ED				
Level 1	1.00	(Reference)	1.00	(Reference)
Level 2	0.93	0.91–0.94	1.02	1.00–1.03
Level 3	0.53	0.51–0.55	0.62	0.58–0.65
Hospital location				
Metropolitan city	1.00	(Reference)	1.00	(Reference)
Provincial area	0.94	0.93–0.95	0.91	0.90–0.93

*EDLOS* Emergency department length of stay, *aOR* adjusted odds ratio, *CI* Confidence interval, *KTAS* Korean triage and acuity scale, *ED* Emergency department, *CCI* Charlson comorbidity index

### Variables associated with in-hospital mortality

The results of the multivariate logistic regression analyses with in-hospital mortality as the dependent outcome are shown in Table 4. After adjusting for patient and hospital variables, prolonged EDLOS was associated with an increased in-hospital mortality risk (aOR, 1.18; 95% CI, 1.16–1.20). artificial ventilation in the ED was a significant risk factor for in-hospital mortality (aOR, 2.73; 95% CI, 2.66–2.80). Age ≥ 65 y gave a nearly two-fold increase in risk for in-hospital mortality (aOR, 1.98; 95% CI, 1.95–2.02), and transfer from another hospital gave a 65% increased risk (aOR, 1.65; 95% CI, 1.61–1.68). In addition, a CCI score of 1 or higher predicted greater in-hospital mortality risk, while a KTAS score of 2 or higher predicted lower in-hospital mortality risk. With regard to hospital variables, hospital staffed bed category and ED type were significantly associated with in-hospital mortality, but contrary to the results of the multivariate analyses for prolonged EDLOS, aOR increased as the number of hospital beds and ED level increased. The risk of in-hospital mortality for critically ill patients admitted to hospitals with < 300 staffed beds was 23% higher than those admitted to hospitals with ≥ 1,000 staffed beds (aOR, 1.23; 95% CI, 1.18–1.27). These findings were consistent with those at the ED level. In the sensitivity analysis, the magnitude and direction of the aOR of patient and hospital variables did not change after excluding patients presented in the level 3 ED (Additional file 1: Table S4).

**Table 4:** Multivariate logistic regression analysis for in-hospital mortality

	In-hospital mortality	
	aOR	95% CI
Patient variables		
EDLOS ≥ 6 h (vs. < 6 h)	1.18	1.16–1.20
Aged ≥ 65 y (vs. < 65)	1.98	1.95–2.02
Female (vs. male)	0.90	0.89–0.91
Insurance type		
National health insurance	1.00	(Reference)
Medical aid	1.09	1.07–1.11
Uninsured or other	1.13	1.09–1.17
Injury-related presentation (vs. no)	0.76	0.74–0.78
Arrival via emergency ambulance (vs. other)	1.50	1.47–1.53
Transferred-in (vs. direct)	1.65	1.61–1.68
Time of presentation		
Day	1.00	(Reference)
Evening	0.91	0.89–0.92
Night	0.88	0.86–0.90
KTAS score		
1	1.00	(Reference)
2	0.34	0.33–0.35
3	0.24	0.24–0.25
4	0.19	0.19–0.20
5	0.26	0.24–0.28
Unidentified	0.25	0.24–0.26
Artificial ventilation in ED (vs. no)	2.73	2.66–2.80
CCI score		
0	1.00	(Reference)
1	1.17	1.14–1.21
2	1.35	1.33–1.38
≥ 3	1.92	1.88–1.97
Season		
Spring	1.00	(Reference)
Summer	0.97	0.95–0.99
Fall	1.03	1.01–1.05
Winter	1.08	1.05–1.10
Year		
2017	1.00	(Reference)
2018	1.05	1.04–1.07
2019	1.01	0.99–1.03
Hospital variables		
Hospital staffed bed		
≥ 1,000	1.00	(Reference)
800–999	1.03	1.00–1.05
600–799	1.10	1.07–1.12
300–599	1.15	1.12–1.18
< 300	1.23	1.18–1.27
Level of ED		
Level 1	1.00	(Reference)
Level 2	1.16	1.14–1.19
Level 3	1.24	1.19–1.28
Hospital location		
Metropolitan city	1.00	(Reference)
Provincial area	0.95	0.94–0.97

*EDLOS* Emergency department length of stay, *aOR* adjusted odds ratio, *CI* Confidence interval, *KTAS* Korean triage and acuity scale, *ED* Emergency department, *CCI* Charlson comorbidity index

**Fig. 2:**
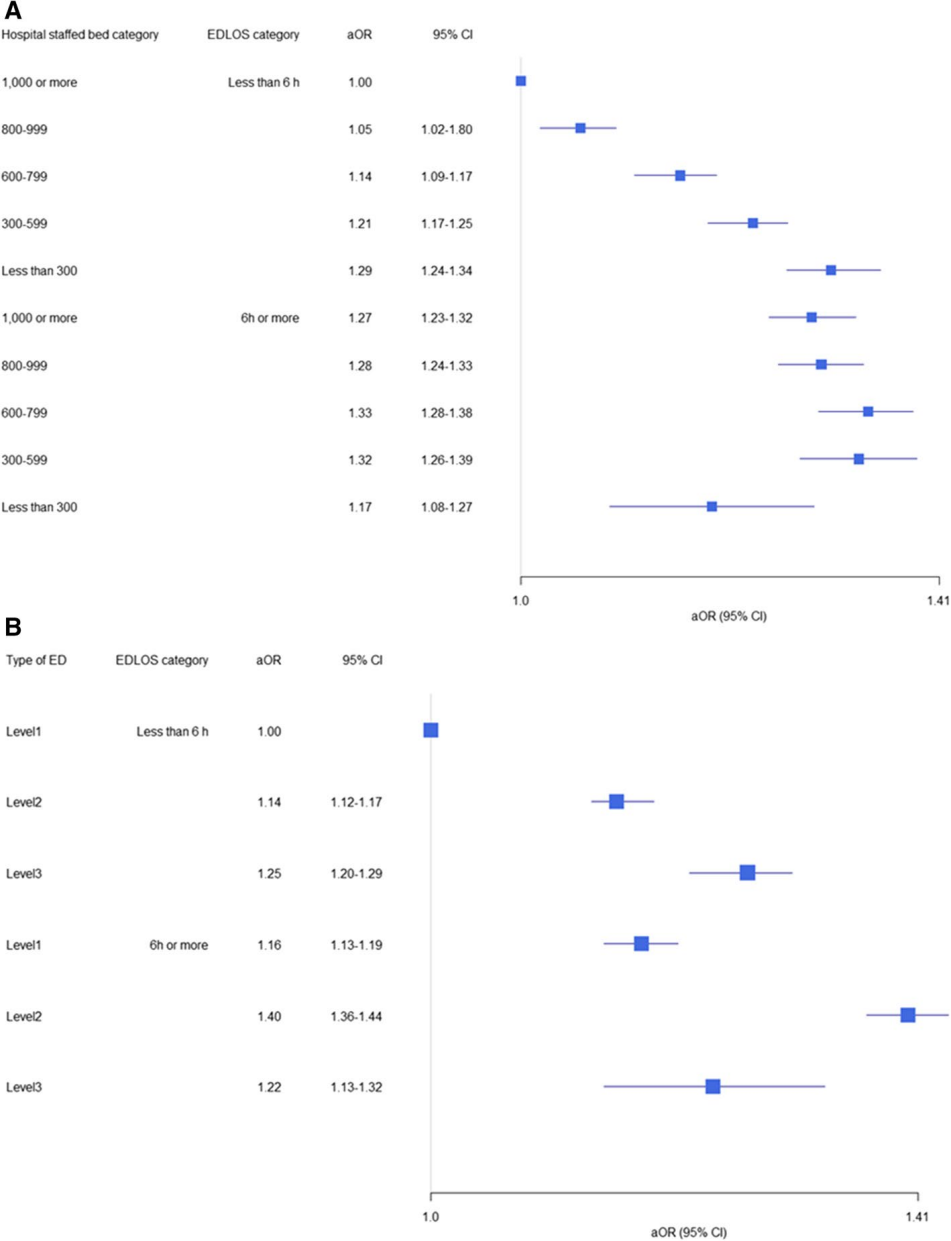
Stratified analysis of in-hospital mortality according to emergency department length of stay and hospital characteristics. Each blue square and line represent the adjusted odds ratio and 95% confidence interval, respectively. (**A)** hospital staffed beds; (**B**) level of ED; EDLOS, emergency department length of stay; aOR, adjusted odds ratio; CI, confidence interval; ED, emergency department

In the stratified analysis, critically ill patients with an EDLOS < 6 h had a higher risk of in-hospital mortality as the staffed beds in the admitted hospital decreased. In contrast, critically ill patients with an EDLOS of ≥ 6 h showed no significant difference in mortality risk depending on the hospital staffed bed categories (Fig. 2-A). These findings were consistent at the ED level (Fig. 2-B).

## Discussion

We conducted a nationwide study, and found a median EDLOS of 3.3 h in critically ill adults admitted directly to the ICU from the ED in Korea. However, 25.3% of these ICU admissions did not meet the criterion of an EDLOS < 6 h, which is an internationally recognised performance indicator used to evaluate the quality of emergency care [25, 37, 38, 49]. Comparing the data reported from other countries, the median EDLOS for critically ill adult patients in Korea was longer than that for Australia (2.5 h) [26], and shorter than that for the US (4–5 h) [3, 6], and Canada (7 h) [29].

The most common primary diagnoses for ED presentation leading to ICU admission were potentially serious cardiovascular, cerebrovascular, and respiratory diseases, and head trauma. However, the top ten primary diagnoses accounted for only 43.3% of all ICU admissions to the ED. Similar to other countries, our finding demonstrates that critically ill patients receiving care in a Korean ED setting represent a highly heterogeneous population [50–52], highlighting the challenges of providing critical care in such an environment [53].

In our study, prolonged EDLOS was significantly associated with night-time presentations, suggesting decreased access to specialist consultations and diagnostic or treatment modalities compared to regular working hours [29, 54]. Patients assigned to the lower acuity scores in the initial triage were more likely to have prolonged EDLOS than those assigned to higher acuity scores. Possible explanations for prolonged EDLOS in patients with lower acuity scores include diagnostic uncertainty requiring additional diagnostic testing and specialist consultations [41], lowering the priority of patients assigned to higher acuity scores [55], and deteriorating clinical condition while the patient is waiting in the ED. Age ≥ 65 y was also associated with prolonged EDLOS. Older patients may have an increased risk of under-triage due to the presentation of non-specific symptoms or vital signs compared with younger patients, which could lead to prolonged EDLOS [56]. In terms of hospital variables, greater numbers of staffed beds and higher ED levels generally represent more in-hospital resources that could increase ED throughput and output. However, the logistic regression model showed an inverse relationship with prolonged EDLOS. According to the input-throughput-output conceptual model, this means that larger hospitals and higher levels of EDs have more “inputs” than smaller hospitals [57]. Indeed, there were more critically ill patients in larger hospitals and in hospitals with higher ED levels, and these patients also had a significantly longer median EDLOS [58, 59].

Here, as in previous studies, prolonged EDLOS was significantly associated with in-hospital mortality [23–28]. In terms of patient variables of interest, logistic regression analysis identified age ≥ 65 y, arrival via emergency ambulance, transfer from other hospitals, night-time presentation, higher initial triage score, artificial ventilation in the ED, and CCI score of 1 or higher as independent risk factors for in-hospital mortality, which is consistent with findings reported in previous studies. Interestingly, even after adjusting for EDLOS and patient variables, the difference in mortality risk between the ED levels and hospital staffed bed categories persisted. As mentioned earlier, hospitals with higher ED levels and more staffed beds cared for more critically ill patients. Increasing evidence suggests that hospitals with higher patient volumes achieve better patient outcomes across various medical conditions and surgical procedures [60–63]. Our findings may reflect this “volume-outcome relationship”. Previous studies have suggested several causal pathways whereby hospital patient volume can affect mortality. First, larger hospitals have more available resources, such as consultants, advanced diagnostic capabilities, and emergency procedural intervention, in order to provide resource-intensive care for specific conditions such as myocardial infarction or sepsis [62]. Second, larger hospitals which deal with higher patient volumes may have greater exposure to time-sensitive conditions, which can lead to the development of institutional policies and treatment processes that improve the quality of care for critically ill patients [64]. However, Nguyen et al. suggested that volume-outcome relationships can be partially mediated by managerial and organizational factors [65]. This view emphasises the importance of introducing mitigation strategies regardless of hospital volume. Recent studies on mitigation strategies have shown that suitable interventions, such as ED-based electronic ICU monitoring systems, streamlined admissions, and ED-based ICUs, can reduce EDLOS or improve clinical outcomes in critically ill patients [53, 66–70].

Our study has several limitations. First, the operational definition of critically ill patients was based solely on ICU admission without objective physiological parameters. The criteria for ICU admission may vary significantly among hospitals. Alternative methodologies for identifying critically ill patients, such as the acute physiology and chronic health evaluation or the simplified acute physiology score, require data not collected in the NEDIS. However, the operational definition used in this study provides a pragmatic representation of ED use in critically ill patients at the nationwide level [3]. Second, since there is no standard risk adjustment method for critically ill patients in the ED setting [53], we attempted to include as many variables as possible in the regression model, but there may be other unaccounted variables contributing to the observed results [71]. In particular, as mentioned above, objective physiological parameters were not included in the analysis, which limits the results related to in-hospital mortality. Also, information on ED overcrowding, staffing, teaching hospital status, ICU capacity, and organizational factors was not reflected in the analysis because these variables fluctuated over time or were not available from the NEDIS. Future work is needed to assess these factors for association with EDLOS and in-hospital mortality. Third, this study was based solely on data from Korea. Regional differences in practices, institutions, and systems can make knowledge transfer difficult; therefore, further studies from other regions and countries are required. Finally, the statistically significant differences observed in this study may be partly due to the large study population size and should be interpreted with caution.

## Conclusions

In Korea, ED is a significant component of the critical care delivery system, from where more than 200,000 adult critically-ill patients are admitted to ICUs annually. Approximately a quarter of these patients stayed in the ED for ≥ 6 h, and prolonged EDLOS was associated with in-hospital mortality. Hospital characteristics were also associated with prolonged EDLOS and in-hospital mortality, after adjusting for patient characteristics. These results highlight the need to introduce mitigation strategies that target potentially modifiable factors, such as the hospital's organizational and managerial elements.

## Supplementary Information

**Additional file 1:** **Table S1.** Characteristics of critically ill patients directly admitted to the intensive care unit from the emergency department by hospital staffed-bed category. **Table S2.** Characteristics of critically ill patients directly admitted to the intensive care unit by type of emergency department. **Table S3.** Sensitivity analysis for prolonged EDLOS. **Table S4.** Sensitivity analysis for in-hospital mortality.

## Data Availability

The sharing of anonymised data from this study was restricted due to ethical and legal constraints. Data contain sensitive personal health information, which is protected under the Personal Information Protection Act in Korea, thus making all data requests subject to institutional review board (IRB) approval. According to the National Medical Center (NMC) IRB, the data that support the findings of this study are restricted to transmission to those in the primary investigative team. Data sharing with investigators outside the team requires IRB approval. All requests for anonymised data will be reviewed by the research team and submitted to the NMC IRB for approval.

## Abbreviations

ED: Emergency department
ICU: Intensive care unit
US: United States
EDLOS: Emergency department length of stay
NEDIS: National emergency department information system
CCI: Charlson comorbidity index
KTAS: Korean triage and acuity scale
ICD-10: International classification of disease 10th edition
IQR: Interquartile range
aOR: Adjusted odds ratio
CI: Confidence interval
