# Supplementary material for: Patient and hospital characteristics predict prolonged emergency department length of stay and in-hospital mortality: a nationwide analysis in Korea
Source: BMC Emerg Med. 2022 Nov 21;22:183. doi: 10.1186/s12873-022-00745-y (PMC9677700; doi:10.1186/s12873-022-00745-y)
Supplement: Supplementary file 1 — Additional file 1: Table S1. Characteristics of critically ill patients directly admitted to the intensive care unit from the emergency department by hospital staffed-bed category. Table S2. Characteristics of critically ill patients directly admitted to the intensive care unit by type of emergency department. Table S3. Sensitivity analysis for prolonged EDLOS. Table S4. Sensitivity analysis for in-hospital mortality. [file 12873_2022_745_MOESM1_ESM.docx]

**Additional file 1**

**Table S1**. Characteristics of critically ill patients directly admitted to the intensive care unit from the emergency department by hospital staffed-bed category.

|  | ≥1,000 | 800–999 | 600–799 | 300–599 | <300 | *P* value |
| --- | --- | --- | --- | --- | --- | --- |
|  | (n=113,419) | (n=155,278) | (n=128,668) | (n=152,294) | (n=152,294) |  |
| No. of institutions (no. of critically ill patients per institution) | 16 (7,088.7) | 28 (5,545.6) | 36 (3,574.1) | 84 (1,813.0) | 209 (728.7) |  |
| EDLOS, Median (IQR), h | 4.9 (2.4–9.4) | 4.2 (2.4–7.8) | 3.9 (2.3–7.1) | 2.8 (1.8–4.3) | 2.1 (1.4–3.1) | <0.0001 |
| Age |  |  |  |  |  |  |
| ≥65 y | 61,006 (53.7) | 86,635 (55.7) | 72,386 (56.2) | 93,138 (61.1) | 73,522 (68.0) | <0.0001 |
| Median (IQR) | 66 (54–77) | 67 (55–78) | 68 (55–79) | 71 (57–81) | 75 (60–83) | <0.0001 |
| Female | 43,299 (38.1) | 60,678 (39.0) | 51,203 (39.7) | 63,849 (41.9) | 49,035 (45.4) | <0.0001 |
| Insurance type |  |  |  |  |  |  |
| National health insurance | 94,551 (83.3) | 130,781 (84.2) | 105,206 (81.7) | 122,536 (80.4) | 83,049 (76.9) | <0.0001 |
| Medical aid | 9,303 (8.2) | 15,845 (10.2) | 15,091 (11.7) | 21628 (14.2) | 19,232 (17.8) |  |
| Uninsured or other | 9,565 (8.4) | 8,652 (5.6) | 8,371 (6.5) | 8130 (5.3) | 5,682 (5.3) |  |
| Injury-related presentation | 21,465 (18.9) | 25,580 (16.4) | 20,999 (16.3) | 20,617 (13.5) | 11,034 (10.2) | <0.0001 |
| Arrival via emergency ambulance | 41,327 (36.4) | 60,542 (38.9) | 61,568 (47.8) | 70,975 (46.6) | 48,987 (45.3) | <0.0001 |
| Transferred-in | 49,823 (43.9) | 67,611 (43.5) | 42,162 (32.7) | 44,494 (29.2) | 26,008 (24.0) | <0.0001 |
| Time of presentation |  |  |  |  |  |  |
| Day | 48,064 (42.3) | 66,227 (42.6) | 55,049 (42.7) | 67,199 (44.1) | 50,006 (46.3) | <0.0001 |
| Evening | 451,34 (39.7) | 62,689 (40.3) | 51,096 (39.7) | 61,308 (40.2) | 44,068 (40.8) |  |
| Night | 20,221 (17.8) | 26,362 (16.9) | 22,523 (17.5) | 23,787 (15.6) | 13,889 (12.8) |  |
| KTAS score |  |  |  |  |  |  |
| 1 | 13,954 (12.3) | 19,372 (12.4) | 12,342 (9.6) | 10,607 (7.0) | 3,978 (3.7) | <0.0001 |
| 2 | 48,616 (42.8) | 59,571 (38.3) | 49,173 (38.2) | 42,949 (28.2) | 16,719 (15.4) |  |
| 3 | 45,245 (39.8) | 66,181 (42.6) | 56,482 (43.9) | 67,839 (44.5) | 30,399 (28.1) |  |
| 4 | 4,801 (4.2) | 95,16 (6.1) | 9,038 (7.0) | 14,207 (9.3) | 12,048 (11.1) |  |
| 5 | 788 (0.7) | 624 (0.4) | 931 (0.7) | 2,048 (1.3) | 2,537 (2.3) |  |
| Unidentified | 15 (0.0) | 14 (0.0) | 702 (0.5) | 14,644 (9.6) | 42,282 (39.1) |  |
| Artificial ventilation in the ED | 9,393 (8.3) | 9,899 (6.4) | 8,052 (6.3) | 6,524 (4.3) | 3,368 (3.1) | <0.0001 |
| CCI score |  |  |  |  |  |  |
| 0 | 77,882 (68.6) | 103,754 (66.8) | 86,690 (67.3) | 102,495 (67.3) | 72,176 (66.8) | <0.0001 |
| 1 | 6,898 (6.1) | 10,962 (7.1) | 8,602 (6.7) | 11,902 (7.8) | 8,441 (7.8) |  |
| 2 | 19,949 (17.5) | 25,639 (16.5) | 21,778 (16.9) | 25,683 (16.8) | 19,068 (17.6) |  |
| ≥ 3 | 8,690 (7.7) | 14,923 (9.6) | 11,598 (9.0) | 12,214 (8.0) | 8,278 (7.7) |  |
| Season |  |  |  |  |  |  |
| Spring | 28,368 (25.0) | 38,597 (24.8) | 31,902 (24.7) | 38,303 (25.1) | 27,582 (25.5) | <0.0001 |
| Summer | 28693 (25.2) | 38863 (25.0) | 32856 (25.5) | 37985 (24.9) | 27238 (25.2) |  |
| Fall | 29,027 (25.5) | 40,056 (25.7) | 33,134 (25.7) | 38,091 (25.0) | 26,303 (24.3) |  |
| Winter | 27,331 (24.0) | 37,762 (24.3) | 30,776 (23.9) | 37,915 (24.8) | 26,840 (24.8) |  |
| Year |  |  |  |  |  |  |
| 2017 | 36,926 (32.5) | 50,430 (32.4) | 41,221 (32.0) | 50,207 (32.9) | 37,848 (35.0) | <0.0001 |
| 2018 | 38,996 (34.3) | 50,760 (32.6) | 42,184 (32.7) | 50,848 (33.3) | 35,731 (33.0) |  |
| 2019 | 37,497 (33.0) | 54,088 (34.8) | 45,263 (35.1) | 51,239 (33.6) | 34,384 (31.8) |  |

Data are presented as number (%), unless otherwise indicated.

ICU, intensive care unit; ED, emergency department; EDLOS, emergency department length of stay; IQR, interquartile range; KTAS, Korean triage and acuity scale; CCI, Charlson comorbidity index

**Table S2.** Characteristics of critically ill patients directly admitted to the intensive care unit by type of emergency department

|  | Level 1  (n=253,879) | Level 2  (n=298,988) | Level 3  (n=104,755) | *P* value |
| --- | --- | --- | --- | --- |
| No. of institutions (no. of critically ill patients per institution) | 39 (6,509.7) | 125 (2,391.9) | 209 (501.2) |  |
| EDLOS, Median (IQR), h | 4.1 (2.3–7.6) | 3.3 (2.0–6.0) | 2.2 (1.5–3.2) | <0.0001 |
| Age |  |  |  |  |
| ≥65 y | 140,379 (55.2) | 175,044 (58.5) | 71,264 (68.0) | <0.0001 |
| Median (IQR) | 67 (55–78) | 69 (56–79) | 75 (60–83) | <0.0001 |
| Female | 98,387 (38.7) | 122,358 (40.9) | 47,319 (45.1) | <0.0001 |
| Insurance type |  |  |  |  |
| National health insurance | 208,359 (82.0) | 247,201 (82.6) | 80,563 (76.9) | <0.0001 |
| Medical aid | 25,744(10.1) | 36,415 (12.1) | 18,940 (18.0) |  |
| Uninsured or other | 19,776 (7.8) | 15,372 (5.14) | 5,252 (5.0) |  |
| Injury-related presentation | 51,329 (20.2) | 41,186 (13.7) | 7,180 (6.9) | <0.0001 |
| Arrival via emergency ambulance | 101,335 (39.9) | 136,875 (45.7) | 45,189 (43.1) | <0.0001 |
| Transferred-in | 111,092 (43.7) | 92,540 (30.9) | 26,466 (25.2) | <0.0001 |
| Time of presentation |  |  |  |  |
| Day | 107,370 (42.2) | 129,624 (43.3) | 49,551 (47.3) | <0.0001 |
| Evening | 102,298 (40.2) | 119,748 (40.0) | 42,249 (40.3) |  |
| Night | 44,211 (17.4) | 49,616 (16.5) | 12,955 (12.3) |  |
| KTAS score |  |  |  |  |
| 1 | 31,841 (12.5) | 26,070 (8.7) | 2,342 (2.2) | <0.0001 |
| 2 | 104,307 (41.0) | 101,188 (33.8) | 11,533 (11.0) |  |
| 3 | 105,724 (41.6) | 139,739 (46.7) | 20,683 (19.7) |  |
| 4 | 11,049 (4.4) | 28,373 (9.5) | 10,188 (9.7) |  |
| 5 | 935 (0.4) | 3,252(1.1) | 2,741 (2.6) |  |
| Unidentified | 23 (0.0) | 366 (0.1) | 57,268 (54.6) |  |
| Artificial ventilation in the ED | 16,642 (6.6) | 17,906 (6.0) | 2,688 (2.6) | <0.0001 |
| CCI score |  |  |  |  |
| 0 | 176,610 (69.5) | 196,748 (65.8) | 69,639 (66.4) | <0.0001 |
| 1 | 16,761 (6.6) | 21,656 (7.2) | 8,388 (8.0) |  |
| 2 | 39,600 (15.5) | 54,088 (18.0) | 18,429 (17.5) |  |
| ≥ 3 | 20,908 (8.2) | 26,496 (8.9) | 8,299 (7.9) |  |
| Season |  |  |  |  |
| Spring | 63,377 (24.9) | 74,683 (24.9) | 26,692 (25.4) | <0.0001 |
| Summer | 64,398 (25.3) | 74,697 (24.9) | 26,540 (25.3) |  |
| Fall | 65,346 (25.7) | 75,585 (25.2) | 25,680 (24.5) |  |
| Winter | 60,758 (23.9) | 74,023 (24.7) | 25,843 (24.6) |  |
| Year |  |  |  |  |
| 2017 | 80,810 (31.8) | 98,720 (33.0) | 37,102 (35.4) | <0.0001 |
| 2018 | 84,957 (33.4) | 98,338 (32.8) | 35,224 (33.6) |  |
| 2019 | 88,112 (34.7) | 101,930 (34.0) | 32,429 (30.9) |  |

Data are presented as number (%), unless otherwise indicated.

ICU, intensive care unit; ED, emergency department; EDLOS, emergency department length of stay; IQR, interquartile range; KTAS, Korean triage and acuity scale; CCI, Charlson comorbidity index

**Table S3. Sensitivity analysis for prolonged EDLOS.**

|  | EDLOS ≥6 h | | EDLOS ≥12 h | |
| --- | --- | --- | --- | --- |
|  | aOR | 95% CI | aOR | 95% CI |
| Patient variable |  |  |  |  |
| Age ≥65 y (vs. <65 y) | 1.17 | 1.16–1.19 | 1.11 | 1.09–1.13 |
| Female (vs. male) | 1.03 | 1.01–1.04 | 1.01 | 0.99–1.02 |
| Insurance type |  |  |  |  |
| National health insurance | 1 | (Reference) | 1 | (Reference) |
| Medical aid | 1.28 | 1.26–1.31 | 1.26 | 1.23–1.29 |
| Uninsured or other | 0.83 | 0.81–0.86 | 0.82 | 0.79–0.86 |
| Injury-related presentation (vs. no) | 0.64 | 0.63–0.65 | 0.69 | 0.67–0.70 |
| Arrival via emergency ambulance (vs. other) | 0.99 | 0.98–1.01 | 0.97 | 0.95–0.99 |
| Transferred-in (vs. direct) | 0.93 | 0.91–0.94 | 1.03 | 1.01–1.05 |
| Time of presentation |  |  |  |  |
| Day | 1 | (Reference) | 1 | (Reference) |
| Evening | 1.05 | 1.04-1.07 | 3.32 | 3.26–3.38 |
| Night | 1.38 | 1.36–1.41 | 2.48 | 2.42–2.54 |
| KTAS score |  |  |  |  |
| 1 | 1 | (Reference) | 1 | (Reference) |
| 2 | 0.87 | 0.85–0.89 | 0.86 | 0.84–0.88 |
| 3 | 1.12 | 1.09–1.14 | 1.00 | 0.97–1.02 |
| 4 | 1.50 | 1.46–1.55 | 1.27 | 1.22–1.32 |
| 5 | 1.51 | 1.40–1.62 | 1.21 | 1.10–1.33 |
| Unidentified | 1.02 | 0.73–1.43 | 0.86 | 0.55–1.34 |
| Artificial ventilation in the ED (vs. no) | 0.89 | 0.87–0.92 | 0.94 | 0.91–0.98 |
| CCI score |  |  |  |  |
| 0 | 1 | (Reference) | 1 | (Reference) |
| 1 | 1.40 | 1.37–1.43 | 1.31 | 1.27–1.35 |
| 2 | 1.32 | 1.30–1.34 | 1.26 | 1.23–1.28 |
| ≥3 | 1.50 | 1.46–1.53 | 1.38 | 1.34–1.41 |
| Season |  |  |  |  |
| Spring | 1 | (Reference) | 1 | (Reference) |
| Summer | 1.05 | 1.03–1.07 | 1.11 | 1.08–1.13 |
| Fall | 0.94 | 0.92–0.95 | 0.90 | 0.88–0.92 |
| Winter | 0.94 | 0.93–0.96 | 0.92 | 0.90–0.94 |
| Year |  |  |  |  |
| 2017 | 1 | (Reference) | 1 | (Reference) |
| 2018 | 1.11 | 1.09–1.12 | 1.10 | 1.08–1.12 |
| 2019 | 1.05 | 1.04–1.07 | 1.00 | 0.98–1.02 |
| Hospital variables |  |  |  |  |
| Hospital staffed beds |  |  |  |  |
| ≥1,000 | 1 | (Reference) | 1 | (Reference) |
| 800–999 | 0.69 | 0.67–0.70 | 0.81 | 0.79–0.82 |
| 600–799 | 0.63 | 0.62–0.64 | 0.73 | 0.71–0.74 |
| 300–599 | 0.24 | 0.23–0.24 | 0.29 | 0.28–0.29 |
| <300 | 0.10 | 0.10–0.11 | 0.17 | 0.16–0.18 |
| Type of ED |  |  |  |  |
| Level 1 | 1 | (Reference) | 1 | (Reference) |
| Level 2 | 0.93 | 0.92–0.94 | 1.02 | 0.99–1.03 |
| Hospital location |  |  |  |  |
| Metropolitan city | 1 | (Reference) | 1 | (Reference) |
| Provincial area | 0.95 | 0.94–0.96 | 0.91 | 0.89–0.92 |

EDLOS, emergency department length of stay; aOR, adjusted odds ratio; CI, confidence interval; KTAS, Korean triage and acuity scale; ED, emergency department; CCI, Charlson comorbidity index

**Table S4. Sensitivity analysis for in-hospital mortality.**

|  | In-hospital mortality | |
| --- | --- | --- |
|  | aOR | 95% CI |
| Patient variables |  |  |
| EDLOS ≥6 h (vs. <6 h) | 1.19 | 1.17–1.21 |
| Aged ≥65 y (vs. <65) | 1.94 | 1.90–1.97 |
| Female (vs. male) | 0.90 | 0.89–0.92 |
| Insurance type |  |  |
| National health insurance | 1 | (Reference) |
| Medical aid | 1.10 | 1.07–1.13 |
| Uninsured or other | 1.17 | 1.13–1.22 |
| Injury-related presentation (vs. no) | 0.77 | 0.75–0.79 |
| Arrival via emergency ambulance (vs. other) | 1.53 | 1.49–1.56 |
| Transferred-in (vs. direct) | 1.63 | 1.59–1.66 |
| Time of presentation |  |  |
| Day | 1 | (Reference) |
| Evening | 0.91 | 0.90–0.93 |
| Night | 0.89 | 0.87–0.91 |
| KTAS score |  |  |
| 1 | 1 | (Reference) |
| 2 | 0.34 | 0.33–0.35 |
| 3 | 0.24 | 0.24–0.25 |
| 4 | 0.20 | 0.19–0.21 |
| 5 | 0.25 | 0.22–0.27 |
| Unidentified | 0.22 | 0.16–0.31 |
| Artificial ventilation in ED (vs. no) | 2.67 | 2.61–2.74 |
| CCI score |  |  |
| 0 | 1 | (Reference) |
| 1 | 1.18 | 1.14–1.22 |
| 2 | 1.32 | 1.30–1.35 |
| ≥3 | 1.94 | 1.90–1.99 |
| Season |  |  |
| Spring | 1 | (Reference) |
| Summer | 1.07 | 1.04–1.09 |
| Fall | 0.97 | 0.95–0.99 |
| Winter | 1.02 | 0.99–1.05 |
| Year |  |  |
| 2017 | 1 | (Reference) |
| 2018 | 1.06 | 1.04–1.08 |
| 2019 | 1.02 | 0.99–1.04 |
| Hospital variables |  |  |
| Hospital staffed bed |  |  |
| ≥1,000 | 1 | (Reference) |
| 800–999 | 1.03 | 1.01–1.06 |
| 600–799 | 1.10 | 1.07–1.13 |
| 300–599 | 1.17 | 1.14–1.20 |
| <300 | 1.21 | 1.16–1.26 |
| Type of ED |  |  |
| Level 1 | 1 | (Reference) |
| Level 2 | 1.16 | 1.14–1.18 |
| Level 3 |  |  |
| Hospital location |  |  |
| Metropolitan city | 1 | (Reference) |
| Provincial area | 0.93 | 0.91–0.94 |

EDLOS, emergency department length of stay; aOR, adjusted odds ratio; CI, confidence interval; KTAS, Korean triage and acuity scale; ED, emergency department; CCI, Charlson comorbidity index
